# The Role of Uncoupling Protein 2 in Renal Adaptation to Hypertension: Mechanistic Insights

**DOI:** 10.3390/biomedicines14102367

**Published:** 2026-10-17

**Authors:** Julia Sperhake, Rolf Schreckenberg, Tsunehisa Sato, Klaus-Dieter Schlüter

**Affiliations:** 1 Physiologisches Institut, Justus-Liebig-Universität, 35392 Giessen, Germany; julia.sperhake@gmx.de (J.S.); rolf.schreckenberg@physiologie.med.uni-giessen.de (R.S.); sato_tsune@outlook.jp (T.S.); 2 Department of Anesthesiology and Intensive Care, Hamamatsu University of Medicine, Hamamatsu 431392, Japan

**Keywords:** pyruvate dehydrogenase, renin–angiotensin system, kidney injury molecule 1 (KIM-1)

## Abstract

**Background/Objectives:** Hypertension leads to chronic kidney disease, and downregulation of mitochondrial uncoupling protein 2 (UCP2) is part of this process. Using UCP2 knockout rats and L-NAME-induced hypertension may identify potential mechanistic insights that couple UCP2 downregulation with renal dysfunction. **Methods:** L-NAME induced hypertension maintained for four weeks in wild-type (WT) and UCP2-depleted rats (KO). Renal function was analyzed by blood urea nitrogen concentration, urinary albumin–creatinine ratio, and urine concentration of the tubule disease marker kidney injury molecule 1 (KIM-1). The expression of genes linked to carbohydrate and fatty acid metabolism, components of the renin–angiotensin system, or oxidative stress were analyzed by qRT-PCR. Key findings were confirmed by Western blot. Oxidative stress was analyzed by dihydroethidium staining. **Results:** In WT and KO rats, L-NAME induced a moderate increase in urine albumin–creatinine ratio and BUN concentration. In KO rats, it also increased the urine concentration of KIM1 and expression of *Havcr*, the gene coding for KIM1. In KO rats, renal expression of pyruvate dehydrogenase was reduced at mRNA and protein level. L-NAME induced the expression of angiotensin receptor type 1 in both strains, but increased the expression of *Ren* only in KO rats. All adaptations were tissue-specific. **Conclusions:** Downregulation of UCP2 accelerates the progression of tubule damage under hypertensive conditions. This effect is linked to metabolic effects shifting oxidative phosphorylation in mitochondria to oxidative glycolysis in the cytosol and furthermore by affecting the expression of genes linked to the local renin–angiotensin system.

## 1. Introduction

Mitochondria are important power packs in cells. They transfer energy from fatty acids and carbohydrates to ATP. This process is described as an electron transport chain and is known as oxidative phosphorylation. The efficiency of this process depends on the presence of uncoupling proteins (UCPs) that reduce the driving force of the electron transport chain, namely the proton gradient. Uncoupling makes the electron transport chain less efficient, but it reduces electron leaks and thereby the formation of reactive oxygen species (ROS) in mitochondria. However, the disadvantage of uncoupling is that less energy from fuel is transferred to ATP, as the energy is lost as heat. There are five different uncoupling proteins known today, named UCP1–5. These different isoforms show a remarkable tissue-specific distribution [1]. Interestingly, the kidney is somehow unique in this aspect as it expresses only one isoform, namely UCP2 [2,3].

The role of UCP2 in basal kidney function or adaptation to stressors such as hypertension, diabetes, ischemia, or inflammation is not well understood. Based on studies using different strains of spontaneously hypertensive rats, most authors described that hypertension induces a reduction in the expression of UCP2 under hypertensive conditions [4,5,6,7,8]. In these models, authors speculated that downregulation of renal expression of UCP2 favored oxidative stress [4,5]. Moreover, several authors also described that the upregulation of UCP2 protected the kidney against hypertension-induced damage [9,10,11]. In contrast, diabetes seemed to upregulate UCP2 expression, which then uncoupled fuel consumption from ATP production, and thereby increased the risk of energy depletion in these kidneys [12,13,14,15]. This suggests that in hypertensive conditions, UCP2 downregulation induces induced oxidative stress in the kidney, whereas in diabetes the upregulation of UCP2 caused energetic stress. Moreover, it is unclear whether UCP2 expression and oxidative stress in cells are coupled. In case of diabetes there are reports that UCP2 depletion increased oxidative stress [16,17], reduced oxidative stress [18], and did not modify the mitochondrial membrane potential and thereby did not modify oxidative stress either [19,20]. In experimental models of sepsis, however, induction of UCP2 reduced oxidative stress and protected the kidney against damage [21,22,23]. Similarly, experimental models of ischemia–reperfusion suggested a protective role for UCP2 [24]. In summary, the data of published studies suggested that UCP2 expression is required for normal kidney function and protects against various stressors. Furthermore, the downregulation of UCP2 under hypertensive conditions is considered a critical point accelerating kidney damage. However, whether lowering renal UCP2 expression affects oxidative stress, cellular metabolism, or interaction with the renin-angiotensin system remains unclear.

In the current study, we used the advantage of UCP2-depleted rats and L-NAME-induced hypertension to investigate the impact of UCP2 on blood pressure, kidney function, kidney metabolism, kidney oxidative stress, and the local renin–angiotensin system.

## 2. Materials and Methods

### 2.1. Animal Subjects

The investigations complied with the *Guide for the Care and Use of Laboratory Animals* of the US National Institutes of Health (NIH publication 85-23, revised 1996). The studies were approved by the local authorities, Regierungspräsidium Gießen (Regional Council of Gießen) approval code: RP Gießen; V54—19 c 20 15 h 01 GI 20/1, G 70/2021, on 1 July 2021.

The study compared four groups of animals (twelve-weeks old female Wistar rats): Wild-type rats (WT) without (WT-C) or with (WT-L) administration of N-ω-nitro-l-arginine methyl ester (L-NAME; 7.5 mg/day in drinking water) and UCP2-depleted rats (UCP2) without (KO-C) or with administration of L-NAME (KO-L). The study used 10 rats per group with randomized exposure to L-NAME or control. In agreement with the local authorities, group sizes were calculated via the resource equation approach. The total number of animals was 20 in two groups (Sham and L-NAME). As this was >10, no animals were added. As a result, the sample of <20 was not unnecessarily large.

To finalize all experiments, rats were weighted and subsequently anesthetized by isoflurane inhalation. After cervical dislocation, kidneys and tibia were separated. Kidneys were weighed and tibia lengths were measured. Subsequently, tissues were quickly frozen in fluid nitrogen and stored until use at −80 °C.

### 2.2. Determination of Blood Pressure

Blood pressures were measured using the non-invasive tail-cuff method (TSE-Systems 209,000 Series, Berlin, Germany) as described before [25]. Before starting the recordings, rats were adjusted to the equipment over one week. A mean of ten consecutive blood pressure readings was obtained for each animal.

### 2.3. RT-PCR

Total RNA was isolated from the kidney using peqGold TriFast (peclab, Biotechnologie GmbH, Germany) according to the manufacturer’s protocol. Genomic DNA contamination was avoided by use of 1 U DNase/µg RNA (Invitrogen, Karlsruhe, Germany). Synthesis of cDNA and subsequent real-time PCR was performed as described before [26]. A list of primers used in this study can be found in the Supplement Table S1. For the screening of our samples, we used reference genes for the following pathways: tubule marker: *Havcr1* and *Acta2*; metabolism: *Pdha1*, *Gapdh*, *Slc2a4*, *Cpt1b*; renin–angiotensin system: *Ren*, *Agtr1*, *Ace1*, *Ace2*; oxidative stress: *Sod2*, *Maoa*, *Maob*, *Cat*, *Sod1*, *Sod3*.

### 2.4. Western Blot

Protein extracts from tissues were generated as described before [26]. Primary antibodies were directed against PDHA1 (GTX104015; GeneTex, Irvine, CA, USA), GAPDH (CB1001; Calbiochem, Merck KGaA, Darmstadt, Germany), renin (PA5-21690; Thermo Fisher Scientific, Dreieich, Germany), ACE2 (NBP1-76614; Novus Biological; Abingdon, UK), and actin (A2668; Sigma, St. Louis, MO, USA). Secondary antibodies (HRP-conjugated) directed against rabbit IgG were purchased from Affinity Biologicals (Ancaster, ON, Canada; P 0448).

### 2.5. Dihydroethidium Staining

Dihydroethidium (DHE) staining was used to quantify oxidative stress as described in [25]. Cryosections of the kidneys were incubated with dihydroethidium (DHE, D23107; Thermo Fisher Scientific, Dreieich, Germany) for 10 min at 37 °C in a light-protected humidity chamber. Subsequently, slides were fixed (Dako fluorescence mounting medium S3023; Dako, North America Inc., USA). Slides were finally imaged by a Keyence microscope (BZ-X800; Keyence, Neu-Isenburg, Germany). Excitation wavelength was 545 nm and emission was recorded at 605 nm. Quantification was performed by ImageJ software (version 1.54 d).

### 2.6. Kidney Function

Kidney function was analyzed by quantification of marker proteins in blood plasma or spot urine as described before [27]. Blood urea nitrogen (BUN) was analyzed with a coulometric detection kit (Arbor Assays, Ann Arbor, MI, USA) in plasma samples diluted 1:10 in sterile water. KIM-1 was analyzed in spot urine (KIM-1 simpleSTep ELISA kit; Abcam, Cambridge, UK). Microalbumin was also quantified in spot urine by rat microalbumin ELISA (Kamiya Biomedical Comp., Tukwila, WA, USA). Creatinine was analyzed in 1:50-diluted spot urine samples (Creatinine PAP; Eberhard Lehann GmbH, Berlin, Germany). Analysis was performed by an ELISA Reader M200 (Tecan, Männedorf, Switzerland) and reads were evaluated by 4PL (myassay.com).

### 2.7. Statistics

Normal distribution of data was validated by Shapiro–Wilk tests. Normal variance was subsequently analyzed using Levene’s est. Subsequently, two-group comparisons were made by Student *t*-tests to calculate the *p*-value. Two sided one-way ANOVA with Student–Newman–Keuls post hoc analysis was used for comparisons of more than two groups (*p* < 0.05 marked by *, #, or combinations thereof). Two-way ANOVA was performed to identify independent effects and their potential interaction with the two variables of treatment (L-NAME administration) and genotype (WT vs. KO) (*p* < 0.05 marked by Greek letters).

## 3. Results

### 3.1. General Effect of Genetic Depletion of UCP2 and K-NAME Treatment on Rats

Before addressing the specific aims of this study, we checked whether genetic depletion of UCP2 in rats successfully represses the *Ucp2* gene expression in the kidney and whether L-NAME induces hypertension affected *Ucp2* expression in wild-type rats. UCP2 depletion strongly reduced the expression of *Ucp2* in rat kidneys (Figure 1A). Moreover, L-NAME induced a strong reduction in *Ucp2* gene expression by 22% (Figure 1A; effect size: 2.149 (0.907–3.347); *p* = 0.000495). Next, we investigated whether *Ucp2* depletion affected blood pressure. Blood pressures were increased in L-NAME-treated rats (Figure 1B). Moreover, the blood pressures of UCP2 KO rats were slightly higher than in WT rats under basal conditions (Figure 1B; +6%; effect size: 1.365 (2.901–0.221); *p* = 0.102). Similarly, blood pressures were higher in UCP2 KO rats compared to WT rats in the presence of L-NAME (Figure 1B; +3%; effect size: 1.137 (2.621–−0.423; *p* = 0.159). Two-way ANOVA identified UCP2 depletion as an independent factor contributing to blood pressure (*p* = 0.028).

Body weight is a very sensitive parameter when the health status of rodents should be addressed. However, body weights were comparable in all four groups (Figure 1C). Similarly, kidney weights did not vary among the four groups investigated in this study (Figure 1D).

### 3.2. Effect of Genetic Depletion of UCP2 and L-NAME Treatment on Kidney Function

L-NAME induced hypertension increased the albumin–creatinine ratio in the urine of wild-type rats by 318% (Figure 2A; effect size 1.001 (2.383–−0.441); *p* = 0.165) and in KO rats by 338% (Figure 2A; effect size 1.065 (2.379–−0.304); *p* = 0.131). Two-way ANOVA identified L-NAME as an independent risk factor (*p* = 0.048).

L-NAME-induced hypertension increased blood plasma BUN concentration, a marker of glomerular filtration. In wild-type rats, blood plasma BUN increased by 10% (Figure 2B; effect size: 0.935 (2.298–−0.409; *p* = 0.177) and in knockout rats by 13% (Figure 2B; effect size: 1.343 (2.709–−0.087); *p* = 0.066). Again, two-way ANOVA identified L-NAME as an independent risk factor for increased blood plasma BUN concentration (*p* = 0.028). As blood plasma BUN correlated with glomerular filtration rate, this suggested a reduced glomerular filtration rate in L-NAME-treated rats. In contrast, UCP2 depletion alone did not affect blood plasma BUN concentration.

Urine concentration of KIM-1 is a marker of tubular damage. It increased in the L-NAME/KO group, with excessive KIM-1 release in some rats (Figure 2C). The large inter-individual variations did not result in differences exceeding a *p* value <0.05. However, mean urinary KIM-1 concentration in in L-NAME/knockout rats was higher than in L-NAME/wild-type rats, showing a large effect (effect size 1.169 (2.501–−0.222); *p* = 0.137). This tendency was also supported by the fact that *Havcr1* gene expression, the gene coding for KIM-1, displayed significant group differences (Figure 2D; Kruskal–Wallis test *p* = 0.049), with significant differences between knockout and L-NAME/knockout rats (effect size 1.635 (2.841–0.379); *p* = 0.010).

Finally, genetic depletion of UCP2 caused an increased renal gene expression *Acta2* (Figure 2E). UCP2 depletion was identified as an independent risk factor for *Acta2* upregulation by two-way ANOVA (*p* = 0.001).

Collectively, the data on renal function showed that L-NAME induced filter damage, indicated by increased albumin–creatinine ratio, and reduced glomerular filtration rate, indicated by increased plasma BUN concentration. In addition, UCP2 depletion caused tubular damage, indicated by increased expression of *Havcr1*, a gene coding for KIM-1 and increased release of KIM-1 into the urine. Finally, UCP2 depletion increased renal expression of *Acta2*.

### 3.3. Effect of Genetic Depletion of UCP2 and L-NAME Treatment on Metabolism

The data so far showed that transcriptional adaptations contributed to the subsequent phenotypical changes in UCP2-depleted rats. As UCP2 is linked to metabolism, we analyzed whether L-NAME, UCP2 depletion, or any combination thereof affected the gene expression of genes linked to metabolism. The analysis showed that UCP2 depletion reduced the gene expression of *Pdha1*, a gene coding for the catalytic unit of the pyruvate dehydrogenase complex (Figure 3A). Two-way ANOVA identified UCP2 depletion as an independent factor related to reduced *Pdha1* expression (*p* < 0.001). Pyruvate dehydrogenase is an enzyme that links glucose and fatty acid metabolism to oxidative phosphorylation in the mitochondria. In contrast to *Pdha1*, the expression of *Gapdh* was not affected by L-NAME, UCP2 depletion, or any combination thereof. UCP2 depletion reduced the expression of the gene encoding for the insulin-dependent glucose transporter GLUT4 (*Slc2a4*, Figure 3C), but not that of the gene coding for the fatty acid transporter *Cpt1b* (Figure 3D).

In conclusion, the data on transcriptional regulation supports the view that UCP2 depletion reduced the oxidative metabolism of carbohydrates, as it reduced the expression of a gene coding for a protein required for oxidative metabolism as well as a glucose transporter.

### 3.4. Effect of Genetic Depletion and L-NAME Treatment on Renin Angiotensin Systems

The kidney expresses most of the components of the renin–angiotensin system (RAS) producing a tissue-specific RAS system. L-NAME decreased the expression of *Ren*, a gene coding for the protease renin in WT rats (Figure 4A; effect size 1.585 (0.524–2.612; *p* = 0.003). However, UCP2 depletion strongly increased the expression of *Ren* independently of L-NAME (two-way ANOVA: *p* < 0.001). In contrast to the regulations of *Ren*, L-NAME but not UCP2 depletion affected the expression of the *Agtr1*, a gene coding for angiotensin receptor type 1 (Figure 4B; two-way ANOVA *p* = 0.004). The kidney also expressed both isoforms of the angiotensin-converting enzyme (ACE), namely *Ace1* and *Ace2*. While neither L-NAME nor UCP2 depletion affected the expression of *Ace1* (Figure 4C), the expression of the *Ace2* gene was reduced in UCP2-depleted rats (Figure 4D; two-way ANOVA *p* = 0.003). Collectively, the data suggested an upregulation of the blood pressure-increasing gene *Ren* together with a downregulation of the blood pressure-protecting gene *Ace2*. This gave a rationality for the genotype-specific increase in blood pressure in these rats (see Figure 1B).

### 3.5. Tissue Specificity of the Effects of UCP2 Depletion

UCP2 is an uncoupling protein that is expressed in multiple tissues. However, each of these tissues has a more or less unique expression profile of the other UCP isoforms. As mentioned in the introduction, the kidney is unique, as it normally expressed only one UCP isoform, namely UCP2. Thus, it remains an open question whether the observed effects of UCP2 depletion on gene regulation were kidney-specific or not.

The effect of UCP2 depletion on *Pdha1* expression was indeed tissue-specific, showing reduced expression in kidney, spleen, and the cortex of the brain, but it was associated with increased expression of *Pdha1* in skeletal muscles (Figure 5A). Similarly, the induction of *Ren* expression in the kidney was highly specific for this organ and not accompanied by a similar expression in the brain (Figure 5B).

### 3.6. Transfer of Transcriptional Regulation to Protein Expression

Basically, immunoblots confirmed the data on gene expression with lower expression of *Pdha1* and *Ace2*, and higher expression of renin in UCP2-depleted rats (Figure 6A–D). However, there was also an unexpected increase in GAPDH expression in UCP2-depleted rats (Figure 6B). Collectively, these data suggested again a shift from oxidative carbohydrate metabolism to aerobic glycolysis and an activation of the renin–angiotensin system in the same rats, although the variations in the semi-quantitative immunoblots did not result in strong levels of probability.

### 3.7. Effect of UCP2 Depletion on Oxidative Stress

In principle, uncoupling proteins can modify mitochondria-dependent oxidative stress. Based on this assumption, depletion of UCP2 should increase oxidative stress. We show here that under conditions of UCP2 depletion, the mitochondria-specific anti-oxidative protein SOD2 was not modified in its gene expression (Figure 7A). Similarly, the oxidative stress inducing the proteins monoamine oxidase A (MAO-A) and monoamine oxidase B (MAO-B) were not affected in their gene expression (Figure 7B,C). However, gene expression of anti-oxidative proteins in the cytoplasm were affected by UCP2 depletion, i.e., *Cat* (Figure 7D), which showed an increased expression in L-NAME-treated rats (two-way ANOVA *p* = 0.033), but a reduced expression in UCP2-depleted rats (two-way ANOVA *p* = 0.021). Similarly, *Sod1* and *Sod3* were significantly more lowly expressed in UCP2-depleted rats.

The overall contribution of the observed changes in the gene expression of anti-oxidative genes to oxidative stress was finally analyzed by DHE staining. As expected, L-NAME increased oxidative stress, but also depletion of UCP2. Unexpectedly, the combination thereof did not lead to an increase in DHE staining (Figure 8).

## 4. Discussion

The kidney is unique in comparison to other tissues as it expresses in its mitochondria only one uncoupling protein, namely UCP2. Although the level of expression is below that seen in the spleen, which is the tissue with the highest expression of UCP2, there must be a reason why this isoform is stably expressed in the kidney. In the current literature, most authors simply suggest a similar function for UCP2 in the kidney to other tissues, ignoring the specific form of expression profile in comparison to other tissues. Furthermore, such a view ignores the fact that different members of the uncoupling protein family may have different functions. The genetic depletion of UCP2 in rats allowed us to investigate more specifically the basal function of UCP2 in the kidney as well as its modification under pressure overload, a main stressor for kidney function in vivo. The data of our study showed that UCP2 is involved in metabolic adaptation of kidney function. UCP2 expression was associated with proper expression of PDHA1, a catalytic subunit of the pyruvate dehydrogenase complex. Moreover, an increased expression of renin in kidneys from UCP2 knockout rats went along with slightly higher blood pressures in these rats. Finally, the depletion of UCP2 in rat kidneys went along with an increased risk of tubule dysfunction, as shown by higher urine KIM-1 concentrations and higher *HAVCR1* gene expression. This is consistent with previous suggestions that UCP2 is required for proper kidney function. It is also in line with histological findings that located the expression of UCP2 in the kidney preliminary in tubule cells [28]. The new finding of our study is that it suggests a coupling between UCP2 expression and renal metabolism. Furthermore, we show for the first time an association between renal expression of UCP2 and renin expression.

We found reduced renal expression of *UCP2* in hypertensive L-NAME-treated rats. It seems that hypertension in general reduced the renal expression of this uncoupling protein. Similar reports were given for different strains of spontaneously hypertensive rats [4,5,7]. Moreover, the extent of renal dysfunction seemed to be related to the expression level of UCP2 [6,8]. To our best knowledge, there was only one report in the literature with L-NAME-induced hypertension that analyzed UCP2 expression [11]. In that study, the mean expression of UCP2 in L-NAME treated rats was reduced compared to untreated controls, but the effect was less strong than in our study. On the other hand, those authors administered L-NAME for two weeks, whereas we administered L-NAME for four weeks, inducing a stronger hypertensive effect. This may explain the stronger effects in our study. A common downregulation of UCP2 in hypertensive rats was remarkable, as it contrasts with the situation with other stressors, such as diabetes, sepsis, or ischemia–reperfusion. In diabetes models, UCP2 was induced [2,14,15,23,24]. Interestingly, in diabetes it seems that UCP2 induction damaged the kidney, whereas under the condition of hypertension the opposite seems to be the case. Noteworthily, not all studies confirmed this general view [18].

Another important finding of our study is that UCP2 depletion affected renin expression and produced a metabolic shift directing carbohydrate metabolism from mitochondria into the cytosol. In general, renal expression of renin was reduced in hypertensive rats [29,30]. Similarly, L-NAME-induced hypertension reduced the expression of renin in the kidney of the L-NAME-treated wild-type rats in this study as well. However, depletion of UCP2 showed a strong induction of renin expression in the kidneys of knockout rats. Interestingly, overexpression of renin was linked to the generation of a Warburg-like effect in H9c2 cells [31]. It may be possible that the induction of aerobic glycolysis, as supported in our model, by downregulation of pyruvate dehydrogenase and induction of GAPDH expression was also the consequence of increased renin expression. This aspect needs more attention in the future. Nevertheless, based on the analysis of recently published papers the following scenario is possible. UPC2 depletion induced the renal expression of renin as shown in our study. Renin induced a metabolic shift in the direction of aerobic glycolysis, as shown before in H9c2 cells and shown here by downregulation of PDHA1. Impaired PDHA1 activity induced the expression of kidney tubular injury, as shown before [32,33]. In our study, urinary secretion of KIM-1 was increased in UCP2-depleted hypertensive rats and *HAVCR1* expression was also increased. In line with this scenario, UCP2 expression in the kidney was most prominent in tubule cells in which PDHA1 expression is also high [28,34]. Any effect of UCP2 depletion on kidney function must focus on these cells. We found induction of the tubule marker KIM-1 by UCP2 depletion, but neither signs of proteinuria nor loss of glomerular filtration. Of note, inhibition of angiotensin receptor type 1 blocked proteinuria. Our study showed in rats with increased urinary albumin showed a higher expression of AGTR1, the gene coding for angiotensin receptor type 1. However, this was linked to L-NAME-induced hypertension and not UCP2 depletion. Furthermore, tubule cells are responsible for the main consumption of energy in the kidney due to the importance of Na-K-ATPase in resorption processes that take place in these cells. Thus, a metabolic shift altering ATP production will affect these cells first.

While the contribution of UCP2 to pathways affecting metabolism and blood pressure may be explained by our own results given here and the current literature as well, the relationship between UCP2 and oxidative stress remains elusive. In our study, increased oxidative stress occurred in kidneys from L-NAME-treated rats and UCP2-depleted rats. These findings were expected and comply with earlier reports as well (i.e., [6]). However, the combination of both stressors nullified the impact on oxidative stress. Interestingly, in L-NAME-treated WT rats, we observed oxidative stress (increased DHE staining), but at the same time the oxidative defense system was induced, as indicated by an increased expression of *CAT*, a gene coding for catalase. In contrast, in L-NAME-treated KO rats, neither the oxidative defense system nor DHE staining was induced. From these observations, we concluded that the upregulation of *CAT* is part of a compensatory mechanism to fight against oxidative stress that is not required in KO rats, as no oxidative stress occurred in these rats. Thus, the surprising finding that L-NAME did not induce oxidative stress in KO rats was most likely not a technical artefact. Having said that, the question arises as to why oxidative stress was absent in KO rats treated with L-NAME. In this context, a study with a breast epithelial cell line lacking mitochondrial DNA might give us a hint [35]. These cells generated a Warburg-like phenotype (oxidative glycolysis instead of oxidative phosphorylation). Given the transcriptional regulation in our study, a similar effect on metabolism must be expected. Interestingly, if UCP2 was downregulated in this cell line, DHE staining as a readout of oxidative stress was reduced. Similarly, genetically manipulated mice with deficiency in both NOS2 and UCP2 expression showed less oxidative stress than mice with reduced NOS2, but normal expression of UCP2 in an encephalomyelitis model [36]. Therefore, although not commonly recognized, there were exceptions from the rule that less UCP2 expression is linked to more oxidative stress reported before. Shifting metabolism from oxidative glucose metabolism in the direction of aerobic glycolysis loss of UCP2 may decrease oxidative stress. Nevertheless, UCP2 depletion damaged tubule function in these rats. This means that oxidative stress did not trigger tubule damage. Alternatively, the effect of UCP2 expression on metabolism rather than that on oxidative stress may trigger the subsequent loss of kidney function.

An unexpected finding of our study was the increased expression of GAPDH protein in UCP2^-/-^ rats that was not seen at the mRNA level. This supports the general finding that UCP2 deletion favored glycolytic pathways, but in addition it also showed that post-transcriptional regulation supported the abovementioned alteration in transcription.

It must be noted that this study investigated the adaptation of the kidney to L-NAME-induced hypertensive stress only in females. It is an ongoing discussion whether females and males differ in several physiological parameters despite a similar anatomic structure, as discussed in detail before [37]. Here, the focus on female rats was chosen, as females develop kidney disease slower than males and may more suited to studying adaptations to stressors. Whether similar adaptations also occur in males requires future studies.

This study has some limitations, but offers also options for future studies. First, most of the analysis was of a retrospective type and requires confirmation in prospective studies in future. By doing this, key findings on protein expression must be confirmed. The study must be extended to male sex, and histological studies are required, i.e., to show in which cells and parts of the kidney the main changes occur. Finally, a direct link between the renin system and metabolic shifts should be addressed in pharmaceutical studies using these rats.

In summary, our study suggests a mechanistic link that explains a pivotal role for UCP2 in regular kidney function under conditions of hypertension in rats. The study underlines the importance of metabolic regulation in stress adaptation.

## 5. Conclusions

From this experimental study using UCP2 knockout rats, we conclude that hypertension-induced kidney damage is at least in part mediated by pressure-dependent downregulation of UCP2 that causes metabolic alteration favoring the oxidative glycolysis instead of oxidative phosphorylation, increasing the expression of renin, and thereby favors tubular damage.

## Figures and Tables

**Figure 1:**
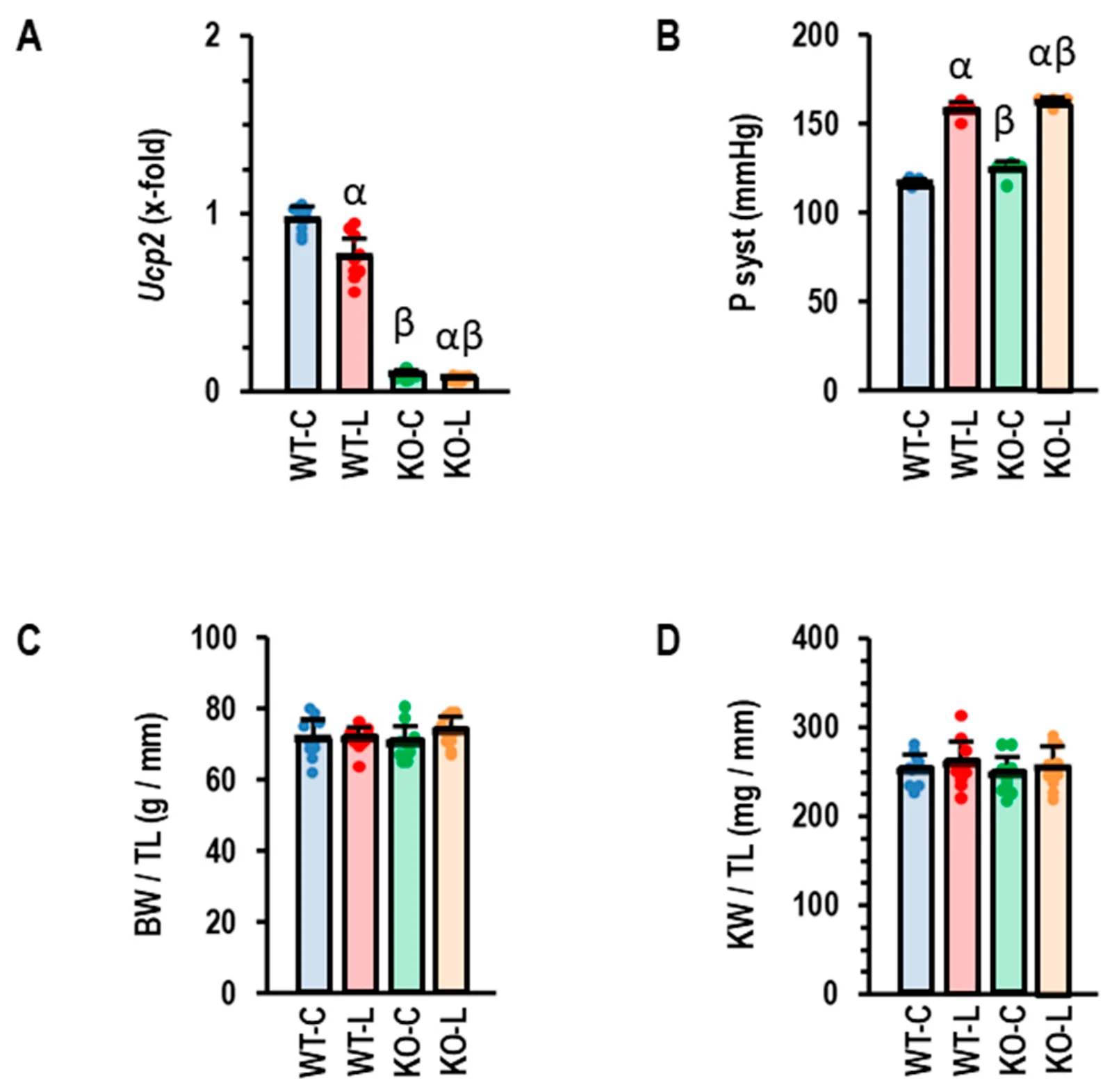
Effects of L-NAME and UCP2 deletion: (**A**) Effect on *Ucp2* expression. (**B**) Effect on systolic blood pressure (P_syst_). (**C**) Effect on body weight (BW). (**D**) Effect on kidney weight (KW). BW and KW are normalized to tibia length (TL). For (**A**,**C**,**D**) n = 10 (each group). (**B**) n = 4 (each group). Data are means ± SD with individual data points given. Two-way ANOVA: α, *p* < 0.05 for treatment. β, *p* < 0.05 for genotype.

**Figure 2:**
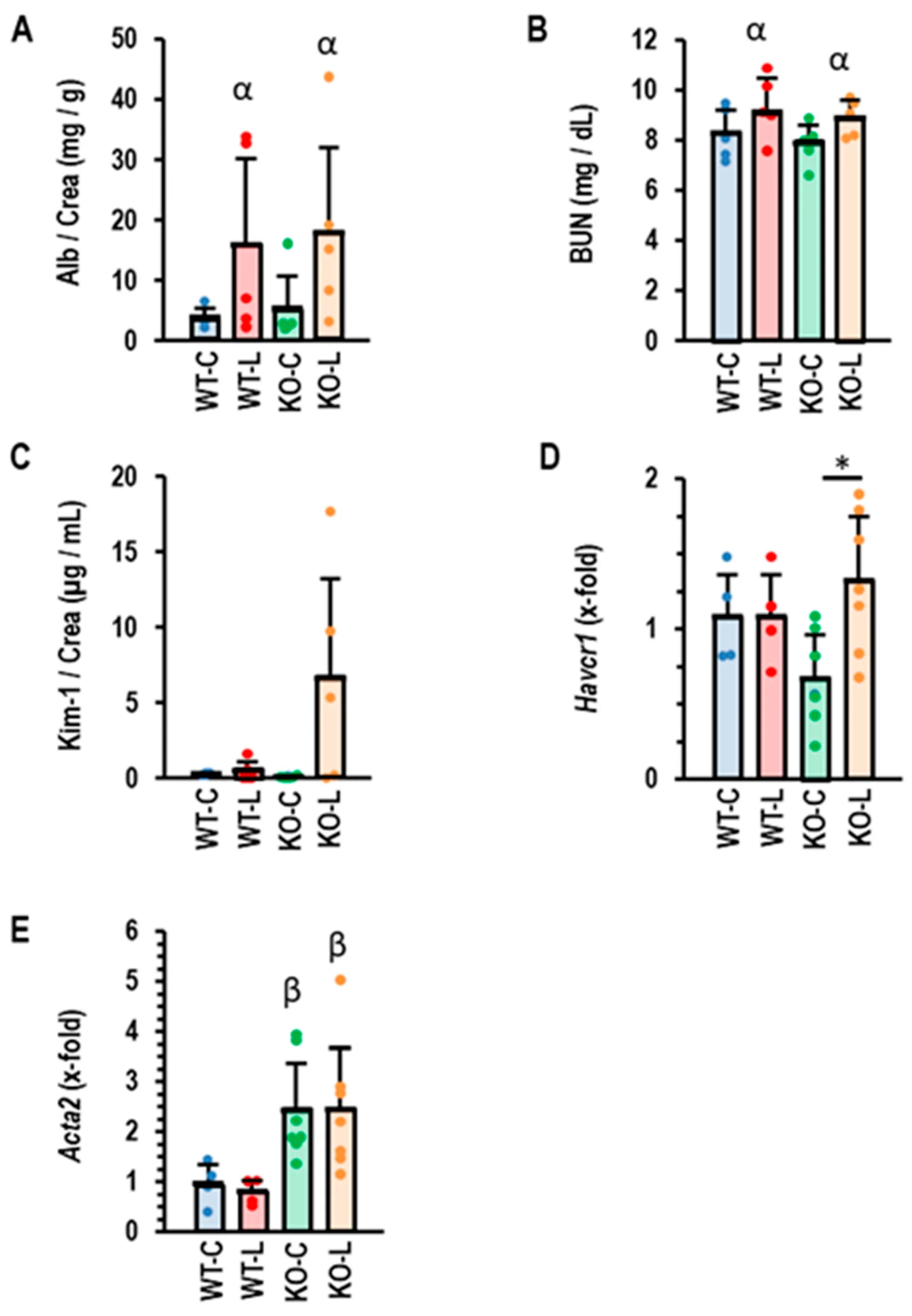
Effects of L-NAME and UCP2 deletion on kidney function: (**A**) Effect on urine albumin concentration normalized to creatinine concentration. (**B**) Effect on blood plasma blood urea nitrogen (BUN) concentration. (**C**) Effect on urine KIM-1 concentration normalized to creatinine concentration. (**D**) Effect on *Havcr1* expression. (**E**) Effect on *Acta2* expression. (**A**–**C**) n = 5 per group; (**D**–**E**) n = 4 (WT) and n = 7 (KO). Data are means ± SD with individual data points given. Two-way ANOVA: α, *p* < 0.05 for treatment; β, *p* < 0.05 for genotype. *; Unpaired two-side *t*-test with *p* < 0.05.

**Figure 3:**
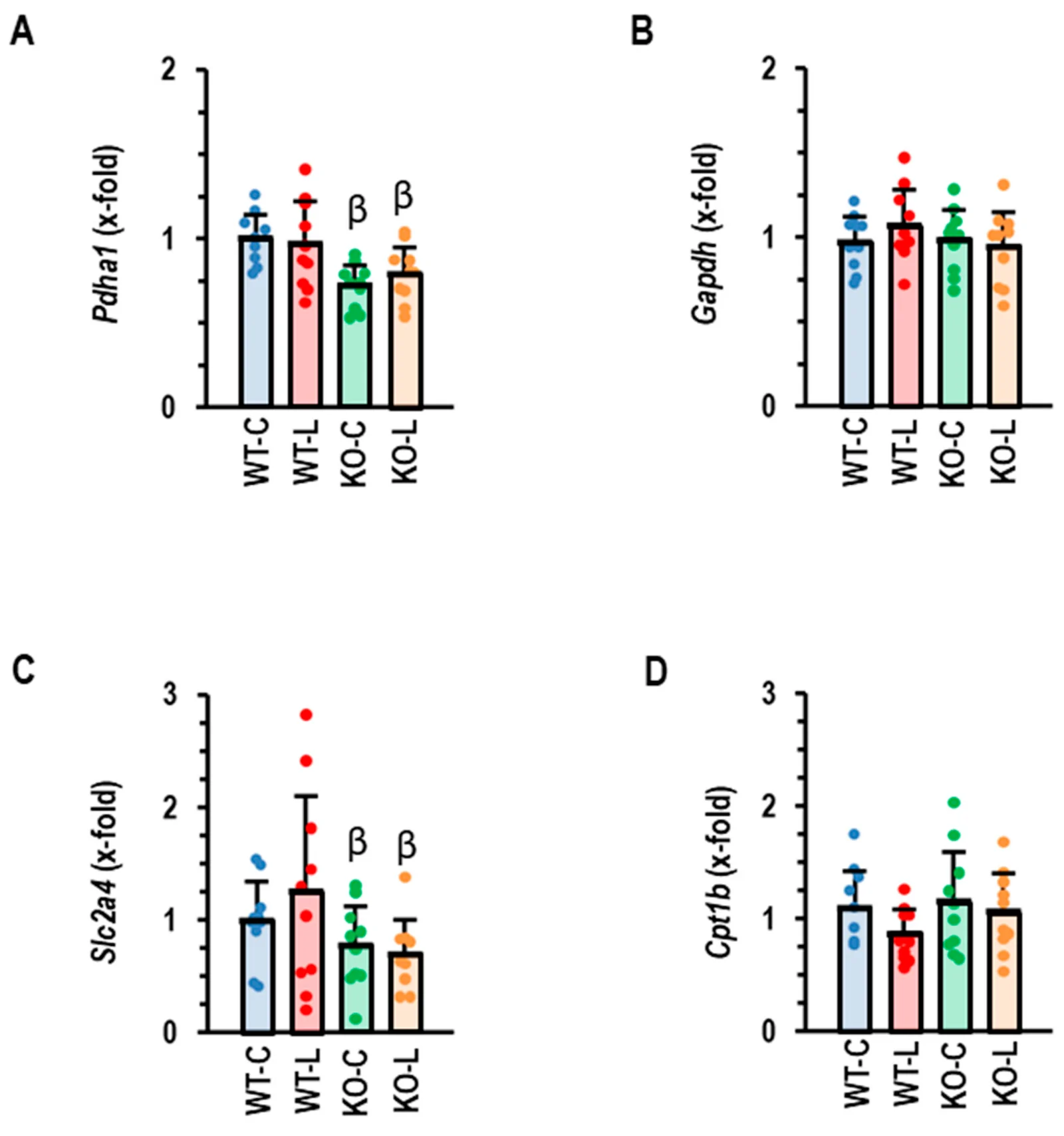
Effects of L-NAME and UCP2 deletion on gene expression of genes coding for proteins involved in metabolism: (**A**) effect on *Pdha1* expression; (**B**) effect on *Gapdh* expression; (**C**) effect on *Slc2a4* expression; (**D**) effect on *Cpt1b* expression. n = 10 per group. Data are means ± SD with individual data points given. Two-way ANOVA: β, *p* < 0.05 for genotype.

**Figure 4:**
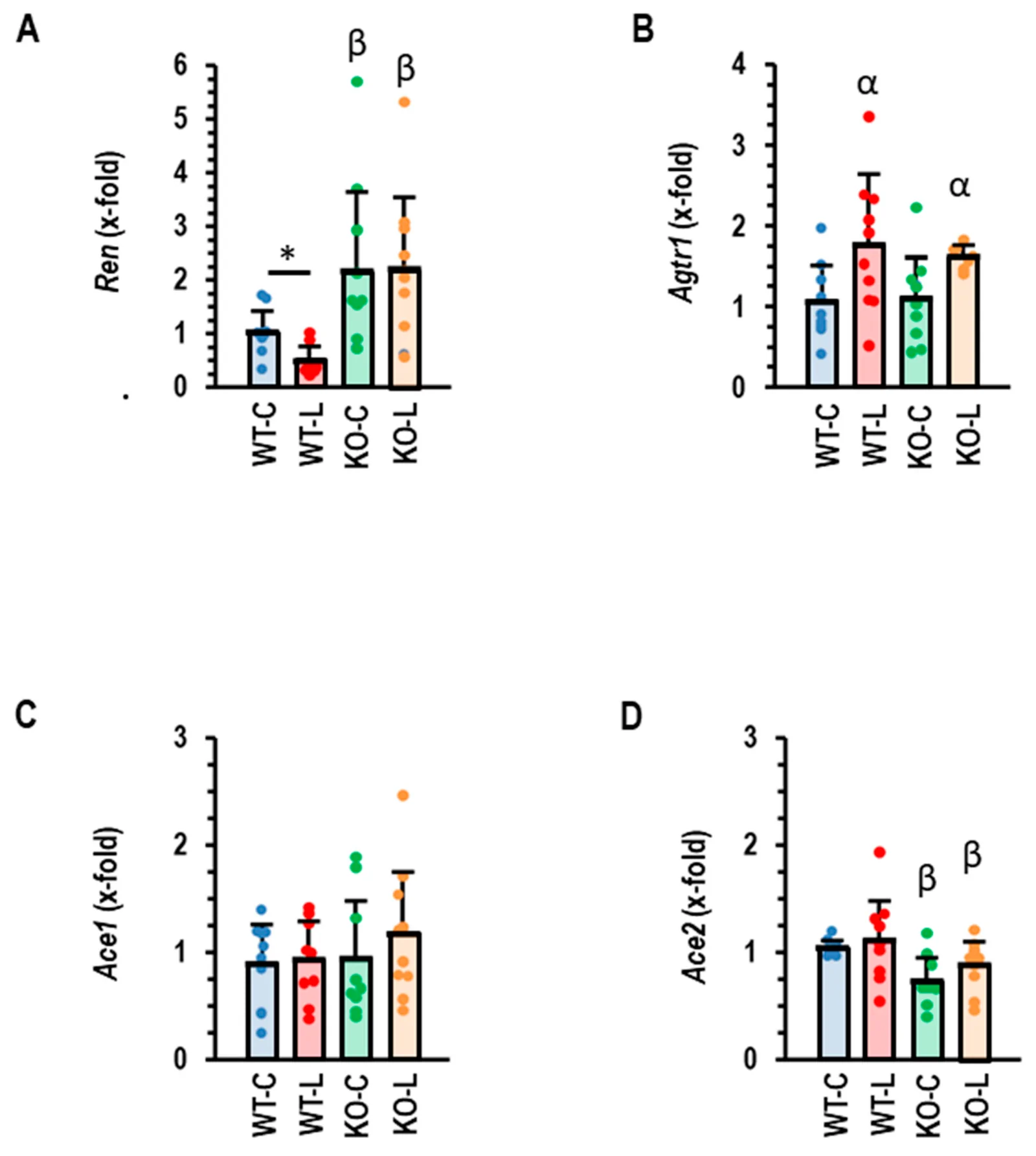
Effects of L-NAME and UCP2 deletion on gene expression of genes coding for proteins involved in blood pressure regulation: (**A**) effect on *Ren* expression; (**B**) effect on *Agtr1* expression; (**C**) effect on *Ace1* expression; (**D**) effect on *Ace2* expression. n = 10 per group. Data are means ± SD with individual data points given. Two-way ANOVA: α, *p* < 0.05 for treatment; β, *p* < 0.05 for genotype. *, *p* < 0.05 between both groups (*t*-test).

**Figure 5:**
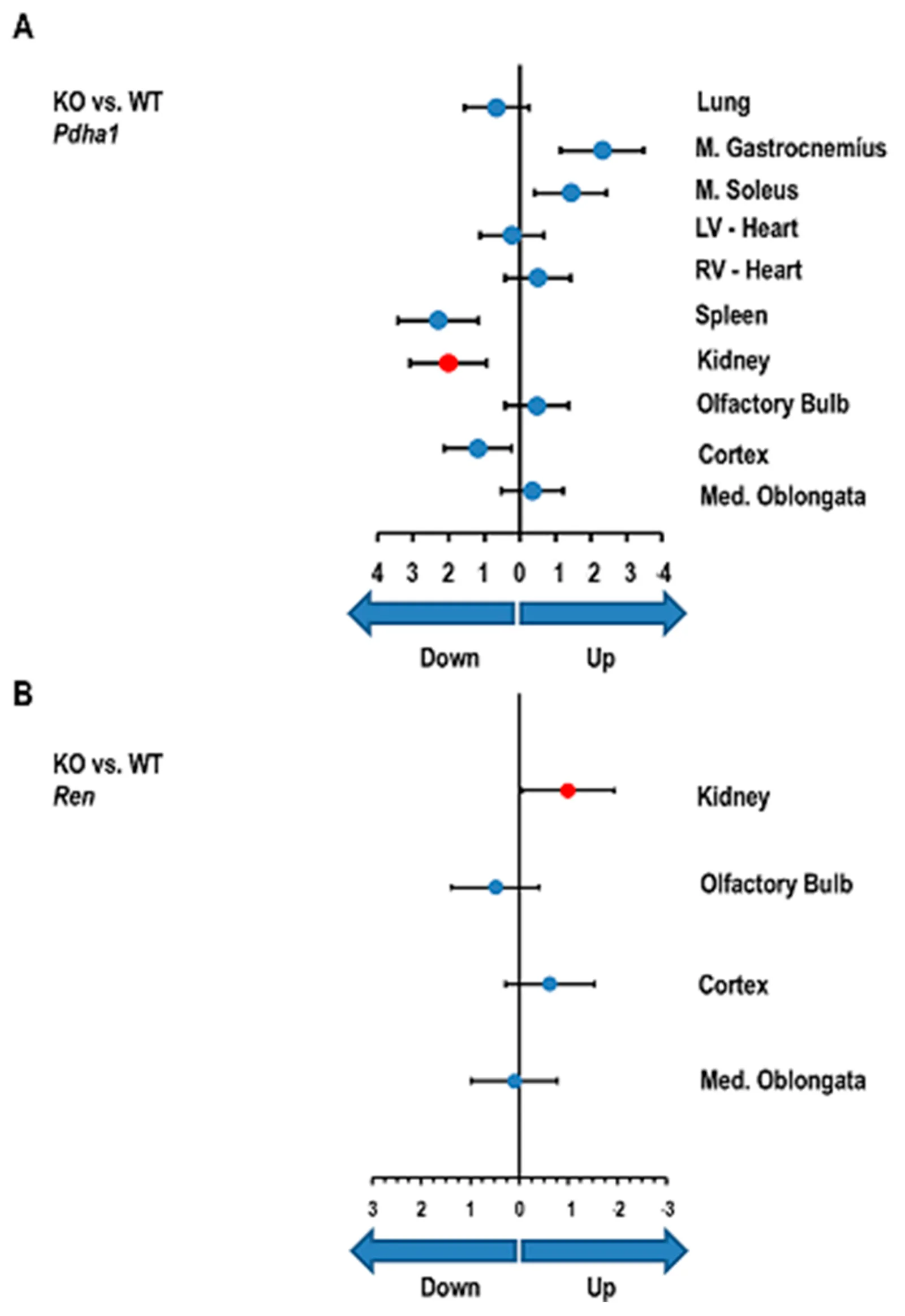
Effects of genetic depletion of UCP2 on the expression of Pdha1 (**A**) and Ren (**B**) in different organs. Data are effect sizes (including 5–95% percentiles). The red dot indicates the kidney. *p* < 0.05 if data points ± percentiles do neither cross nor attach the zero line.

**Figure 6:**
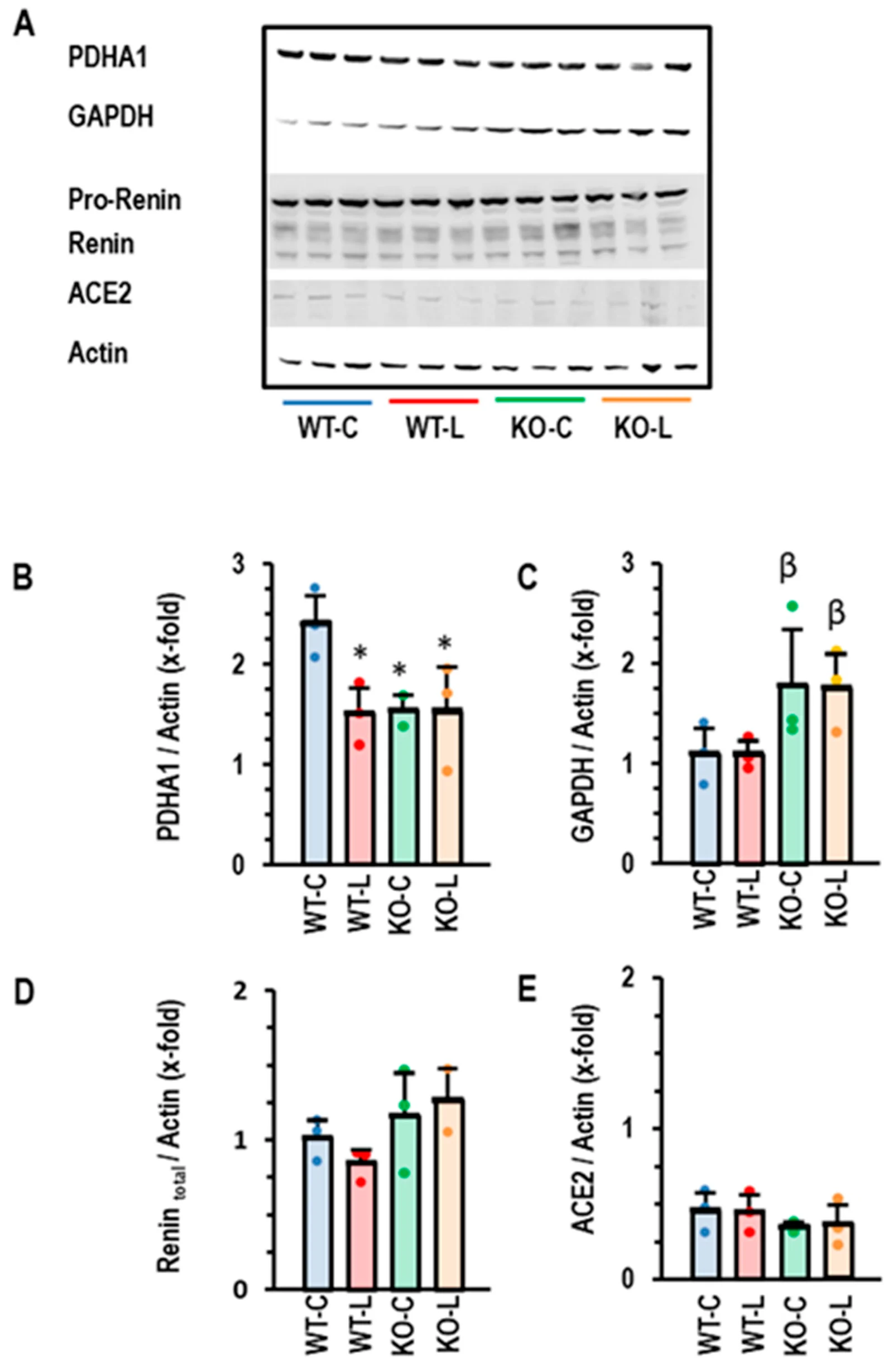
Western blot quantifying the renal expression of PDHA1, GAPDH, renin, and ACE2. (**A**) Original Western blot; (**B**–**E**) quantification of the blot shown in A with means ± SD (n = 3 each group). One-way ANOVA with Student–Newman–Keuls post hoc analysis: *; *p* < 0.05 vs. WT-C. Two-way ANOVA with β, *p* < 0.05 for genotype.

**Figure 7:**
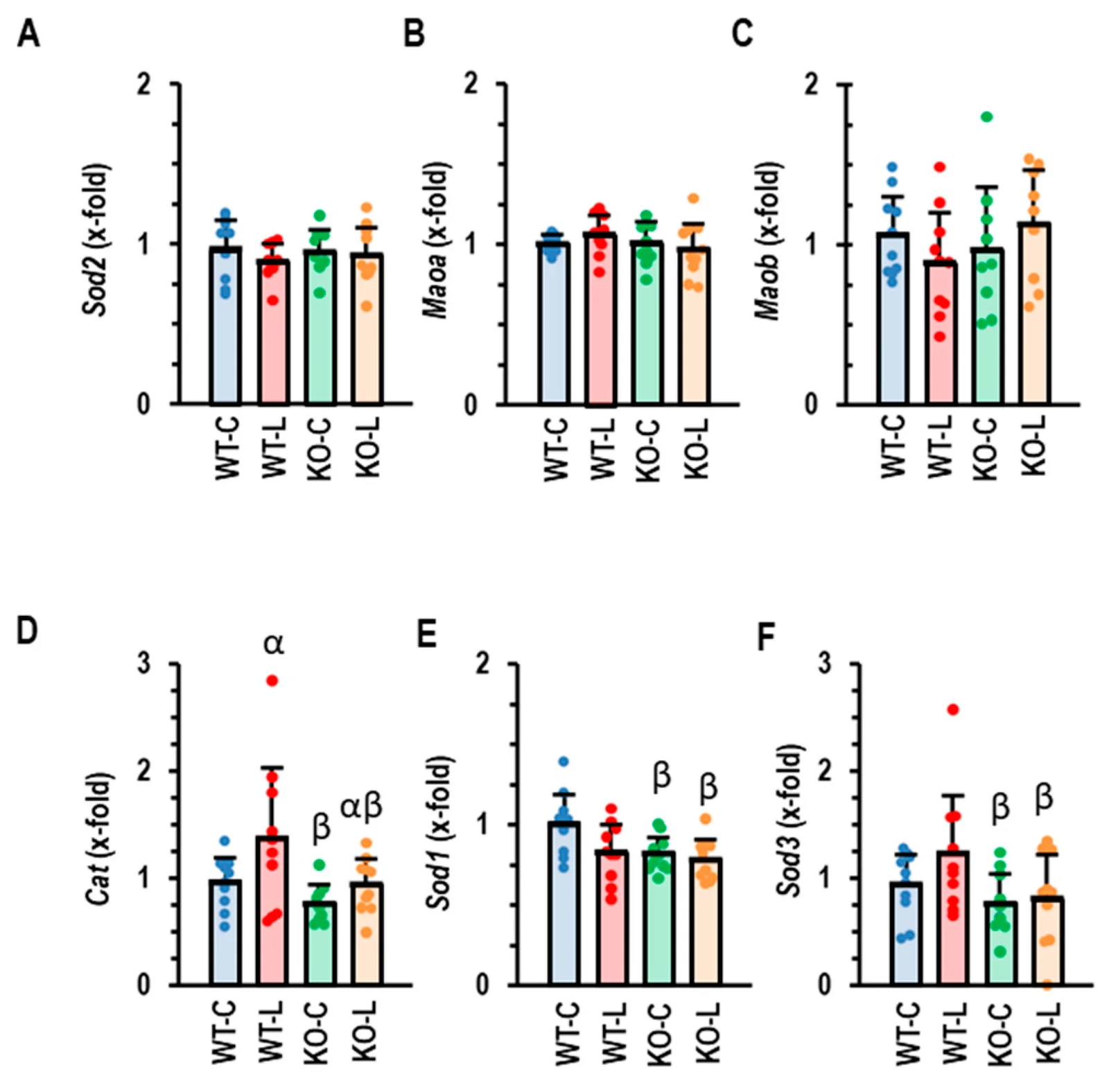
Effect of UCP2 depletion and L-NAME-induced blood pressure on renal expression of genes linked to oxidative stress. (**A**–**F**) Expression of *Sod2*, *Maoa*, *Moab*, *Cat*, *Sod1*, *Sod3*. Data are means ± SD from n = 10 samples per group with original data points. Two-way-ANOVA: α, *p* < 0.05 for treatment; β, *p* < 0.05 for genotype.

**Figure 8:**
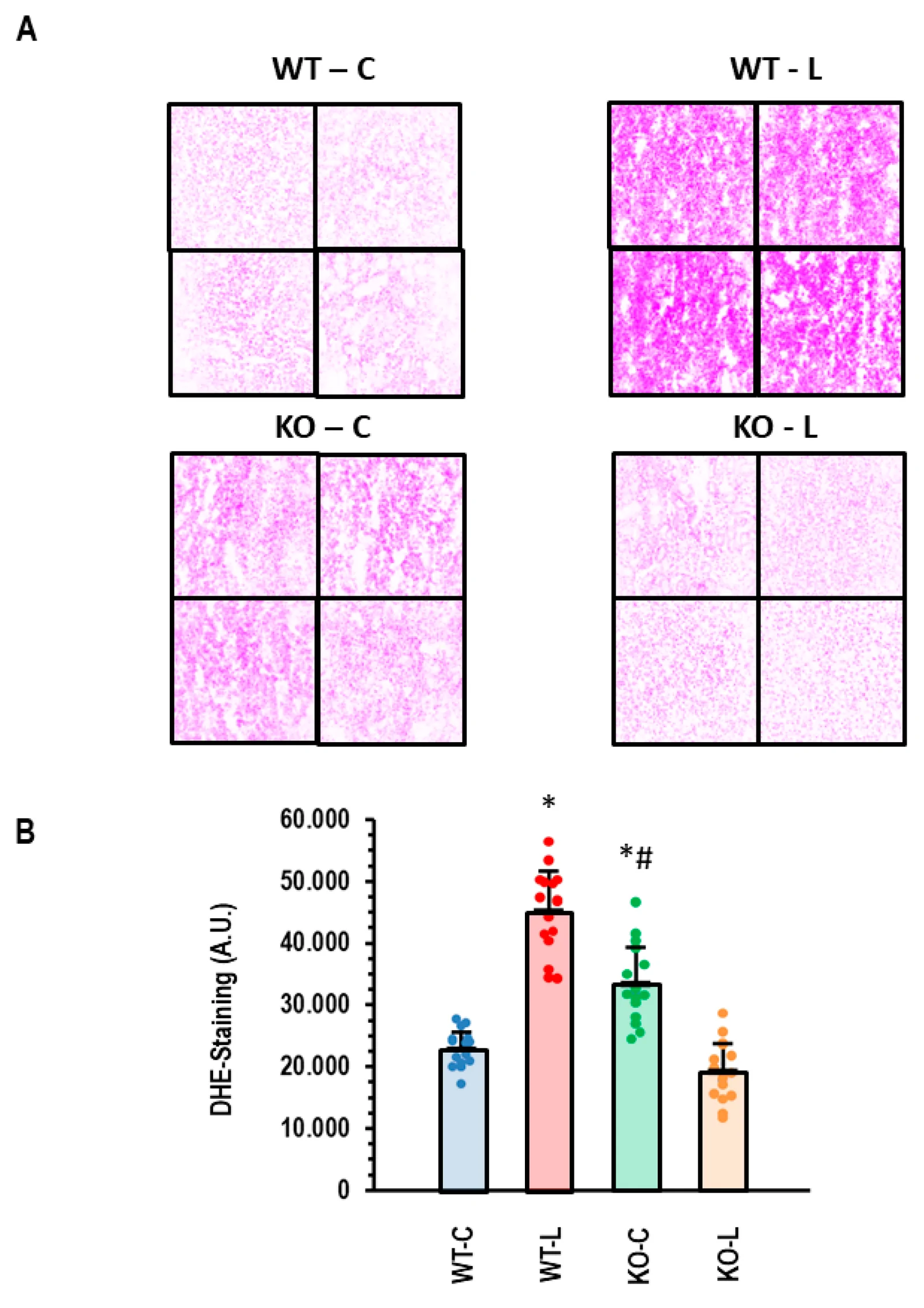
Effect of UCP2 depletion and L-NAME-induced blood pressure on oxidative stress. (**A**) Representative picture. Black bars correspond to 100 µm. (**B**) Quantification of analysis (16 individual data sets from n = 2 animals per group). One-way ANOVA with Student–Newman–Keuls post hoc analysis. *, *p* < 0.05 vs. WT-C; #; *p* < 0.05 vs. WT-L.

## Data Availability

The data presented in this study are available on request from the corresponding author.

## Supplementary Materials

The following supporting information can be downloaded at https://www.mdpi.com/article/10.3390/biomedicines14102367/s1. Table S1: Primers used in this study.
