# Supplementary material for: The Role of Uncoupling Protein 2 in Renal Adaptation to Hypertension: Mechanistic Insights
Source: Biomedicines. 2026 Oct 17;14(10):2367. doi: 10.3390/biomedicines14102367 (PMC13702226; doi:10.3390/biomedicines14102367)
Supplement: Supplementary file 1 [file biomedicines-14-02367-s001.zip › Supplement Table S1.pdf]

Supplement Table S1: List of primers used for PCR

Gene name	forward	reverse	NCBI Ref. Seq.
ACE1	ACAGCTATAACTCGAGTGCCG	ACAGCTCCTTGGCCTTCTTG	NM_012544.1
ACE2	ACAATTGTTGGAACGCTGCC	CGCTTCATCTCCCACCACTT	NM_001012006.1
ACTA2	ACCATCGGGAATGAACGCTT	CTGTCAGCAATGCCTGGGTA	NM_031004.2
AGTR1	GAAGTCTCGCCTTCGCCGCA	CAGCCCTATGGGGAGCGTCG	NM_030985.4
B2M	GCCGTCGTGCTTGCCATTC	CTGAGGTGGGTGGAACTGAGA C	NM_017087
CAT	TTTTCACCGACGAGATGGCA	CTGACTCTCCAGCGACTGTG	NM_012520
CPT1B	GACCCAAAGCAGTACCCCAA	GCAGGGCGTTCGTTTCTGA	NM_013200.2
GAPDH	CTTCTCTTGTGACAAAGTGGACA	CTCGCTCCTGGAAGATGGTG	NM_017008.4
HAVCR1	CTTTGAGGAAGCCGCAGAGA	ATGTTGTCTTCAGCTCGGGG	NM_008767666.2
HPRT	CCAGCGTCGTGATTAGTGAT	CAA GTC TTT CAG TCC TGT CC	NM_012583
MAOA	GTTGGACAAAAATGCTCGGG	GGAACCCACAGGGCAGATACC	NM_033653.1
MAOB	GGCTGTGCTGCTATAATGFFA	ATAATGGACTGGCTGCAGAGC	NM_013198.1
PDHA1	CAGAAGCCGGCAAGCAG	ATACAGCTGATCCGCCTTGAG	NM_001004072.2
REN	GGAGGATGCCTCTCTGGGCACT	GATTTCCCGGACCGAGGGCA	NM_022177.3
SLC2A4	ACCGTCTTCACGTTGGTCTC	ATCAAGATGGCACAGCCACA	NM_012751.1
SOD1	AAGGCCGTGTGCGTGCTGAA	ACATGGAACCCATGCTCGCCT	NM_017050
SOD2	ATGTTGTGTCGGGCGGCGTG	TCGCGTGGTGCTTGCTGTGG	NM_017051
SOD3	TGCTGCCTCCCGATCAGCCA	CCCTGGCTCAGGTCCCCGAA	NM_012880
UCP2	CACCGTCATTGCCTCCCCCG	CGGAGCATGGTCAGGGCACA	NM_019354.2
